# Smart Cupboard for Assessing Memory in Home Environment

**DOI:** 10.3390/s19112552

**Published:** 2019-06-04

**Authors:** Franks González-Landero, Iván García-Magariño, Rebecca Amariglio, Raquel Lacuesta

**Affiliations:** 1 Edison Desarollos, 44002 Teruel, Spain; gonzalezfranks@edisondesarrollos.es; 2 Department of Software Engineering and Artificial Intelligence, Complutense University of Madrid, 28040 Madrid, Spain; 3 Harvard Medical School, Harvard University, Boston, MA 02115, USA; ramariglio@mgh.harvard.edu; 4 Massachusetts General Hospital, Boston, MA 02114, USA; 5 Department of Computer Science and Engineering of Systems, University of Zaragoza, 44003 Teruel, Spain; lacuesta@unizar.es; 6 Instituto de Investigación Sanitaria Aragón, University of Zaragoza, 50009 Zaragoza, Spain

**Keywords:** IoT, memory loss, e-healthcare, Alzheimer’s, door sensors

## Abstract

Sensor systems for the Internet of Things (IoT) make it possible to continuously monitor people, gathering information without any extra effort from them. Thus, the IoT can be very helpful in the context of early disease detection, which can improve peoples’ quality of life by applying the right treatment and measures at an early stage. This paper presents a new use of IoT sensor systems—we present a novel three-door smart cupboard that can measure the memory of a user, aiming at detecting potential memory losses. The smart cupboard has three sensors connected to a Raspberry Pi, whose aim is to detect which doors are opened. Inside of the Raspberry Pi, a Python script detects the openings of the doors, and classifies the events between attempts of finding something without success and the events of actually finding it, in order to measure the user’s memory concerning the objects’ locations (among the three compartments of the smart cupboard). The smart cupboard was assessed with 23 different users in a controlled environment. This smart cupboard was powered by an external battery. The memory assessments of the smart cupboard were compared with a validated test of memory assessment about face–name associations and a self-reported test about self-perceived memory. We found a significant correlation between the smart cupboard results and both memory measurement methods. Thus, we conclude that the proposed novel smart cupboard successfully measured memory.

## 1. Introduction

Dementia is the progressive loss of cognitive functions due to brain damage or disorder. Among dementia types, one of the most well-known and widespread diseases is Alzheimer’s disease, and the number of people who will suffer from this disease is estimated to reach 131.5 million by 2050 [1]. This disease hampers daily life activities such as recognizing faces and remembering names, places, and positions [2]. Potentially 131.5 million people could put themselves at risk if they start developing these symptoms without the proper cautionary measures and palliative treatments.

There is no cure for Alzheimer’s disease, but there are palliative treatments. Some of these are medicines, but none of them has been proven to stop the progression of this disease [3]. Other examples are psychosocial interventions, and these involve stimulation-oriented treatments with art, music, animals, or other recreational activities; however, the efficacy of these treatments remains uncertain [4]. The last of the palliative treatments is caregiving, which is probably the safest one, but it has a great impact in health economics for the necessary resources—mainly caregivers, but also measurement of the state of the disease for suitable caregiving, tagging items in houses, and the maintenance of feeding tubes in the case of eating problems.

On the other hand, the literature claims that Alzheimer’s disease represents the main cause of neurodegenerative dementia in the population aged over 60 years old, with an estimated prevalence of 5–7% [5]. People increase the probability of starting to suffer from Alzheimer’s disease when getting older without noticing. If anyone suffers from Alzheimer’s disease, they need to receive some treatment—the sooner the better.

At present, most people live surrounded by technology, including Internet of Things (IoT) sensor objects that can collect, pre-process, and analyze continuous streams of data (e.g., weather, traffic, finance, and health data). One of the most common goals of the IoT is to enhance the quality of life. IoT technology can track the interactions between quotidian objects and persons, or even among objects, contributing to the digitalization of the physical world. Another goal of the IoT is to connect and synchronize traditional utensils through the Internet in order to deliver a service more efficiently. In this way, all elements that used to connect in a close circuit are now connected through a network, increasing their utility [6]. IoT sensor systems now allow the connection of physical objects so that remote services can be provided through the Internet by analyzing the data from these sensors. Thus, once the hardware of the IoT sensors is installed, programmers can provide new functionalities by developing new software based on different analyses.

In this paper we present a novel sensors system based on the IoT aimed at detecting memory losses for the early detection of some neurodegenerative diseases, by continuously assessing the memory of the user and notifying them when appropriate. Among other features of the system, we can highlight that this measurement method does not require any additional effort from the user, and is continuous. Users only need to go about their daily routine. The sensors system was installed in a cupboard, converting it into an IoT smart cupboard (SC). We used a normal cupboard, such as one that most readers could find at their home or in their kitchen. The SC had some door sensors connected to a Raspberry Pi, programmed to analyze the signals and measure the memory.

The main contribution of the current work over the related works of other authors is its presentation of a low-cost solution that can monitor users in their daily lives for measuring memory with the potential of detecting diseases with memory impairments, without needing qualified staff. In addition, the mechanism is novel, as this is the first work that presents a novel SC for this purpose, and is based on magnetic door sensors with very low prices. This work extends our previous work about IoT collaboration exemplified with a SC prototype [7]. The contribution of the current work over the previous one lies in the use of a more advanced three-door SC prototype that measures memory. This wa proved with experimental results obtained from 23 participants in which the SC measurements correlated with a validated memory test and another test about self-perceived memory.

The current work is organized as follows. The next section reviews the existing related works considering the common technologies in this field. Section 3 describes the design of the proposed SC to assess memory and all its features. Section 4 describes the conducted experiments and the user tests for validating the system. Section 5 presents the main results. Finally, Section 6 discusses the results, draws conclusions, and depicts some future lines of research.

## 2. Related Work

The research community is actively involved in the topic of this work due to the consequences of memory losses on the wellbeing of patients and their social environments. The goal is to reduce their economic impact on the society due to treatment costs. IoT technology allows the interconnection of small low-cost devices practically anywhere. These devices can monitor health indicators and the behaviors of people. There are many works and projects on this topic, and this section introduces the most relevant ones.

Some projects present solutions involving Alzheimer’s disease and Raspberry Pi. For instance, Nonavinakere et al. [8] developed a system that recognizes a person’s face and tells the user the name of that person and the relationship they have with them. The tool was developed thinking about users with Alzheimer’s disease. The system was tested using three different platforms; one of them was a Raspberry Pi 3, and although it was not the fastest platform, it was the most accurate, since it was able to detect a person within certain limits. Crema et al. [9] proposed an embedded platform-based system for early detection of Alzheimer’s disease through transcranial magnetic stimulation (TMS). TMS is a non-invasive way to stimulate the cerebral cortex in order to address Alzheimer’s disease. This system was formed by a magnetic stimulus generator, an electric stimulus generator, a field-programmable gate array, and a Raspberry Pi. The goal was to introduce an alternative technique that supported the early detection of Alzheimer’s with reduced costs, and provided results that were suitable for medical interpretation. Narendiran et al. [10] developed a cognitive assistance system for smart homes. The main aims of the project were (a) to simulate the progression of Alzheimer’s-type dementia by evaluating performance in the execution of an activity of daily living and (b) to provide support for impaired people who need help in daily activities such as preparing a cup of coffee. The system used a camera connected to Raspberry Pi. The camera provided live images to the Raspberry, whose contents were the patient inside the home environment. While the Raspberry was receiving images, it assessed the performance of a task and provided feedback to the patient about their performance. For instance, when a patient forgot some step of some task, the system reminded the patient about this step. Ishii et al. [11] designed an early-detection system for dementia using the Machine-to-Machine (M2M)/IoT platform. The system was formed by sound sensors, motion sensors, pressure sensors, an Arduino board per sensor, a Raspberry Pi board, an M2M server, and the corresponding analysis software. The authors assessed several activities and behaviors of a person inside their home. Several sensors were set up in the home environment, including outdoors near the home and inside some rooms (e.g., bedrooms, bathrooms). Each Arduino board connected to a sensor sent information to the Raspberry Pi. This had two functions; the first was to send information to the M2M server, and the second was to analyze the collected information through the analysis software in order to determine early symptoms of Alzheimer’s disease. It is worth highlighting the mixed use of Arduino and Raspberry Pi boards. Kristalina et al. [12] kept in mind one of consequences of memory impairment, which is that a certain person can forget where they are. In order to address this handicap, they developed a system that involved a Raspberry Pi and an iBeacon. This was a tracking system for patients with memory impairment. Each patient carried an iBeacon device that was responsible for sending the ID and signal strength to the Raspberry Pi and then to the server in order to convert the information to a distance. Due to the amount of noise, it was possible that the obtained distance data did not match with current patient position, so the authors applied the Kalman method in order to estimate the distance between devices. This system was assessed at the Dr. M. Soewandhie hospital, and their tests showed an average percentage measurement error of 7.01% in the actual patient position. Chavan and Chavan [13] proposed a novel system for fall detection in elderly people. They benefited from the new features of Raspberry Pi 3 with respect to the previous version (i.e., Wi-Fi connection) for the creation of a new system with wearables. The system was formed by a laptop, a Raspberry Pi 3, accelerometers, a heart rate sensor, and a temperature sensor. The sensors sent information to the Raspberry, and this determined if there had been a fall. If so, a text message was sent to a mobile device. Paul et al. [14] described a low-cost system for monitoring patients, which was formed by several sensors, a Raspberry Pi, a database, and an application. The system’s aim was to collect patient data (e.g., electrocardiogram signal, blood pressure signal, heart rate signal, blood oxygenation, temperature) in order to send them to the patient’s doctor. The system was set up in Bangladesh with success.

Although they do not make use of a Raspberry Pi, the following projects addressed Alzheimer’s disease with other IoT elements. Chong et al. [15] proposed a system in order to predict a potential Alzheimer’s medical condition. They used a room with movement sensors inside it and analyzed the data obtained from 20 elderly persons by means of five sensors over the course of six months. The results provided three key factors in order to perform a prediction: excess activity levels, sleeping patterns, and repetitive actions. These factors were useful for predicting the early warning signs of Alzheimer’s, and allowed the authors to provide recommendations to caregivers based on the prediction analyses. Navarro et al. [16] developed a fuzzy adaptive cognitive stimulation therapy generation system for Alzheimer’s patients. The aim of the system was to reduce the cognitive burden of care workers and therapists. The system assessed patient behavior through several activities and even through their voice tone and their phrases. This system used the Mente Activa software, whose aim was to provide computer-assisted cognitive therapy. The authors demonstrated the enhancement of patients with the experiments with their system. Finally, Roopaei and Jane [2] focused on another aspect related to memory loss, which was the ability to recognize familiar faces. The authors developed a platform to support patients who suffered from face perception impairment with an assistive intelligence device. The system included an algorithm that recognized a face among entries in a face dataset. The algorithm used deep learning to recognize patterns in faces and match them. Regarding IoT, the authors proposed to use glasses in order to let the user know who was in front of them as well as their relationship.

Table 1 depicts the main differences and similarities of the current work with the most related ones. As one can observe, the current work is the only one that has all the following four features at the same time: (a) it does not need qualified staff, and hence anybody can use it without previous experience; (b) it has the potential to measure memory by just analyzing the daily activities of users; (c) it is a low-cost solution for monitoring; and (d) it can detect symptoms of memory disease in early stages. The most similar work is the one by Ishii et al. [11], as it also has the potential to measure memory and conduct the early detection of memory-impairment related diseases by analyzing daily activities without requiring qualified staff. Even though, the current work has all these features, it is also low cost, thanks to the novel mechanism based on a SC with very low-cost magnetic door sensors.

Considering all the related works presented in this section, we also noticed a gap in the literature about using pieces of IoT-enabled furniture for monitoring the memory of users for the early detection of memory-impairment diseases. The current approach covers this gap in the literature by presenting an IoT SC, built with low-cost magnetic door sensors, introduced in the next section.

## 3. Smart Cupboard for Assessing Memory

**Figure 1:**
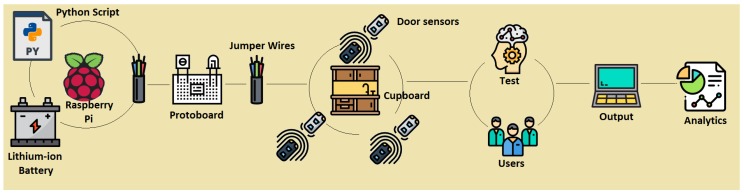
Overview of the smart cupboard and the experiments.

**Figure 2:**
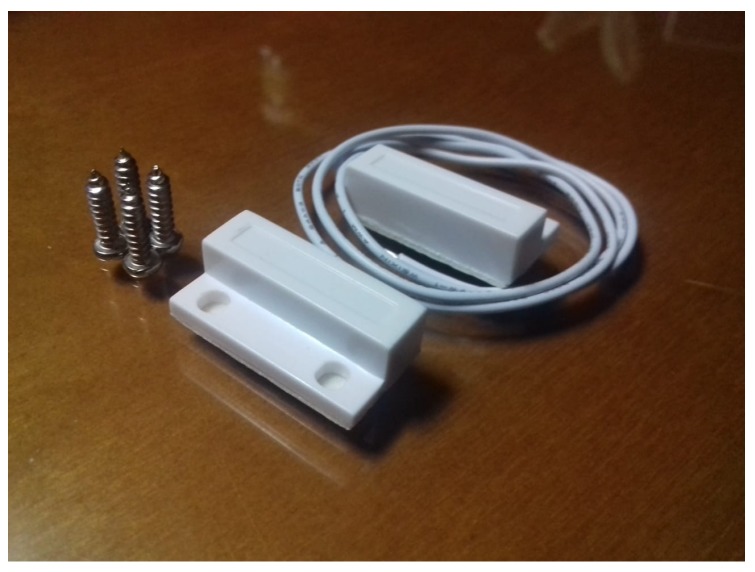
Door sensor.

In Figure 1, the reader can observe a picture that depicts the overall experiment described in this paper. This paper also presents the design of the SC, the assessment method of the SC, and the experimentation with users. The core of the system is formed by a Raspberry Pi model 3 B+, with CPU 1.4 GHz 64-bit quad-core ARMv8, 1 GB Memory (SDRAM) (shared with GPU), 17x GPIO and HAT ID bus, 5 V through MicroUSB or a GPIO header. A lithium-ion battery accompanies the Raspberry Pi, which facilitated the setting up of the system inside the cupboard for the experiments, since a wire connected to power was not necessary. This provided the possibility of installing the sensors system in a cupboard without needing a nearby socket. The autonomy of the battery was 9 h, and we considered that this was enough to assess our system in controlled environments with users. The SC also had magnetic door sensors. The cupboard selection was made based on certain features. The requirements that the furniture must have according to our controlled experiments were (a) to be placed in a kitchen, (b) to have three compartments of the same size (to avoid memory techniques based on the size of objects and compartments), and (c) that each compartment could hold 5 to 10 items without overlapping (to facilitate the acquisition phase based on observation). We used an Excellway MC-38 wired magnetic alarm system door window sensor switch with screw provided by Banggood. Figure 2 shows this magnetic door sensor. Each sensor was composed of two parts; one was attached to the cupboard structure and connected to the Raspberry Pi, and the other was attached to the door, such that both parts were together when the door was closed and apart when it was open. Each sensor closed the circuit when both parts were together (or very near to each other). For our system, we used three pairs of sensors and these were set up in the three doors of the cupboard. A protoboard and jumper wires were used to connect the Raspberry Pi and door sensors, and the schematic design is presented in Section 3.1. We wrote a Python script in order to manage the proposed SC. The script assessed the memory of users based on the analysis of the signals of door sensors, with the algorithm described in Section 3.2.

### 3.1. Schematic Design of the Smart Cupboard

**Figure 3:**
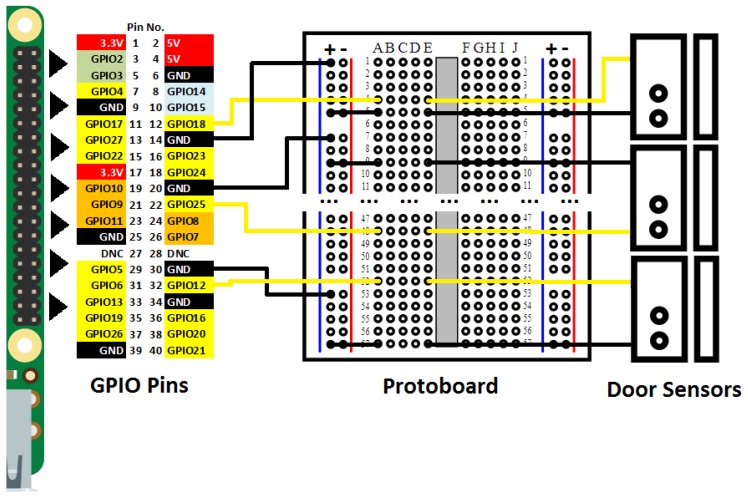
Schematic design of the smart cupboard. GPIO: general-purpose input/output.

The door sensors and the Raspberry Pi were connected through jumper wires and a protoboard, and this section explains in detail how these elements were connected. The Raspberry Pi had a pin series placed on a side of the board, called GPIOs (general-purpose inputs/outputs). They performed multiple input/output operations for different purposes. The Raspberry Pi model used in this work had 40 pins. Figure 3 depicts the schematic design of the SC, indicating the used pins. This schematic design uses the following color notation to distinguish the different pin types:

- Red pins: power to 3.3 V and 5 V.
- Green pins: Communication through Inter-Integrated Circuit (I2C) protocol in order to communicate with peripherals that use this protocol.
- Blue pins: Connection for the universal asynchronous receiver–transmitter (UART) for a conventional serial port.
- Black pins: Connection to ground.
- Orange pins: Communication through the Serial Peripheral Interface (SPI) protocol in order to communicate with peripherals with this protocol.
- White pins: Reserved pins.
- All GPIO pins: Apart from their particular function, all GPIO pins have general-purpose inputs/outputs.

Each door sensor had two wires and, due to their specification, one of these wires needed to be connected to ground and the other one needed to be connected to some input. Pins GPIO 18, GPIO 12, and GPIO 25 were chosen as input pins; pins GPIO 14, GPIO 20, and GPIO 30 were selected as ground pins. These choices were mainly arbitrary, and we only considered that chosen pins with inputs/outputs had a ground pin next to them. Because wires of door sensors could not be directly connected to the Raspberry Pi, we connected these through a protoboard by means of jumper wires. As one can observe in the schematic design, all ground pins were connected through jumper wires to the positive power line of the protoboard (representing this connections with black lines). Then, in order to ensure the connection continuity until the door sensors, a jumper wire was placed from the power line to the central segment of the protoboard for each sensor. In the schematic design, these connections are represented with black lines that go from the “+” column to the “A” column. Finally, door sensors were connected to the circuit through column “E”. It was necessary to use one or several jumper wires in this last step, depending on the distance from the protoboard to each door sensor. Input GPIO pins were directly connected to the protoboard through the central segment, then door sensors were connected with them through the same central segment (represented with yellow lines).

### 3.2. Algorithm for Measuring Memory Based on the Door Sensor Signals

The algorithm was designed to determine when a user finds an item, and when the user searches for an item without success. One research question was: how do we know that the user had success in searching an item inside the SC? In order to answer this question, we had to explain all possible cases in which a user can find an object. The first case is that the user finds a certain item in the first attempt. The second case is that the user finds a certain item in a certain number (denoted as *N*) of attempts, assuming N≥2. The last case is the one in which the user did not find the item.

**Figure 4:**
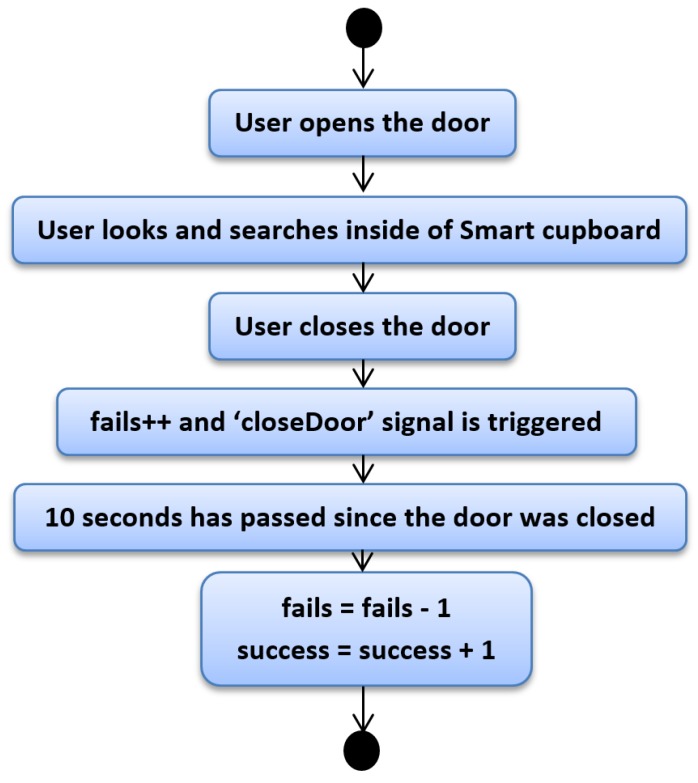
Python script—First case.

Figure 4 depicts the first case. One of the rules that allows understanding this topic is that the user usually has success in finding objects except in some cases. Nonetheless, we do not know how many times the user has attempted to find a certain object. In this first case, the user is going to find a certain object at the first attempt. With the open door of the SC, the user looks and searches for the desired object. Once the user grabs the object, they close the door and at this moment, our script takes note of one fail with the search. It seems illogical that our system would increase the fail counter, but since we do not have another mechanism to determine exactly whether the user has grabbed the desired item or to know whether the user has found the desired item (assuming this reduced set of low-cost sensors), our system marks one fail. However, we kept in mind that if the user does not open another door or the same door in a reasonable amount of time, the most probable reason is that the user found the item. We established 10 s as reasonable amount of time, and hence once the door of the SC was closed and 10 s passed, we estimated with high probability that the user had the desired item. When the door is closed, besides increasing the fail counter, the “closeDoor” signal is triggered, which starts a time counter that allows us to know whether the user opens a SC door in a reasonable time or not. Since we are explaining the case in which an object is found at the first attempt, this time the counter is not interrupted. In following cases we will explain what happens when this signal is interrupted. So, when the system notices that after 10 s the user has not opened any door, it removes a fail from the fail counter and increases a unit on the success counter.

**Figure 5:**
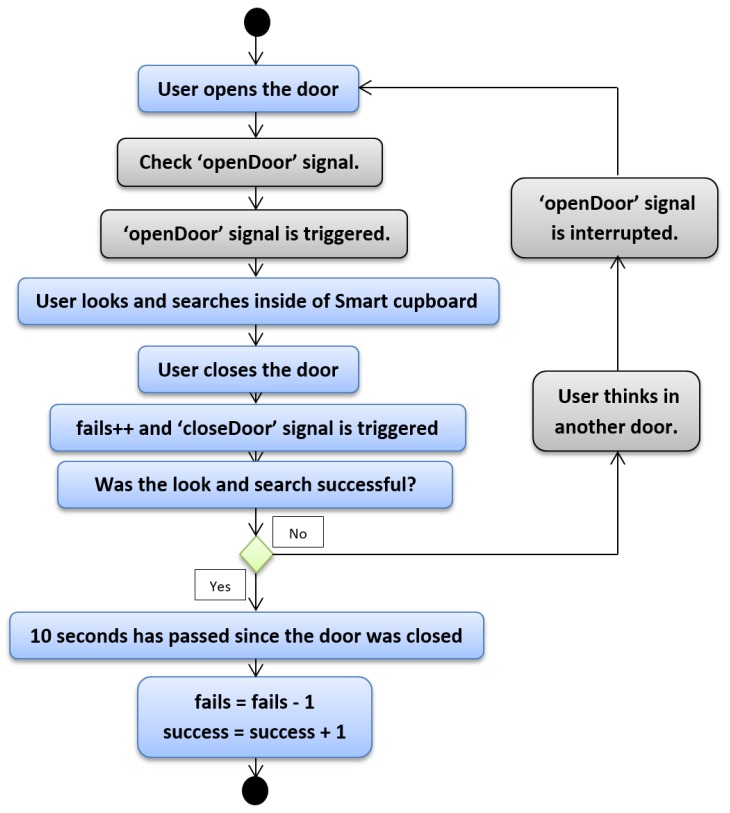
Python script—Second case.

The second case is more complex than the first one. We will explain the same steps as above, but with more signals and certain features. In the second case, the user finds a certain item after *N* attempts. The beginning is the same as the previous case, as one can see in Figure 5, which depicts the diagram of the second case. The user opens the door, and before they search or find anything, the “openDoor” signal is triggered. The aim of this signal is to determine whether another door has been opened before, and in the positive case our system cancels the count of 10 s in order to avoid reducing the fail counter and to prevent increasing the success counter. In this way, our system counts all fails during the process of searching for an item. Focusing again on this second case, we take for granted that the “openDoor” signal has not been triggered, since it is the first time that the user opened the door. Nonetheless, the action of opening the door is the trigger of the “openDoor” signal. The same signals as in the previous case are also triggered, but we have omitted them in this description in order to ease comprehension.

When the “openDoor” signal is launched, the user starts searching for the item, then they close the door. The fail counter increases and the “closeDoor” signal is triggered, and until this point, all steps are the same as in the first case. Now, the user knows whether they have the correct item. If so, it is the first case; if not, the “openDoor” signal must be interrupted. Empirical tests made by ourselves and other users allowed us estimate that 10 s was enough time for someone to be able to open another door of the SC; if not, again, it was assumed that the user found the correct item. Hence, in the second case when the user thinks about what their next door choice will to be and opens it, the “openDoor” signal is interrupted and the cycle starts again until the user opens the correct door. Until this moment the user has always had success in searching for their object, regardless of whether it was found in their first attempt or in attempt *N*. However, in the following case the user does not have any success, since according to our point of view the user does not reach to find the required object. We kept the third case in mind in order to manage two issues: the first is that the user searches for an object but forgets what they were searching for. This situation gives us information about the health of the user’s memory. The second is that in this way we can avoid that someone cheated during the experimentation phase (i.e., if we did not control the opening time of a door and a certain object was not inside the SC, the user would have unlimited time to think about what other compartment the object may be in). In the experimentation phase, this case did not appear at any moment, but it is important to check for this issue because it makes the measurement of memory loss more accurate.

**Figure 6:**
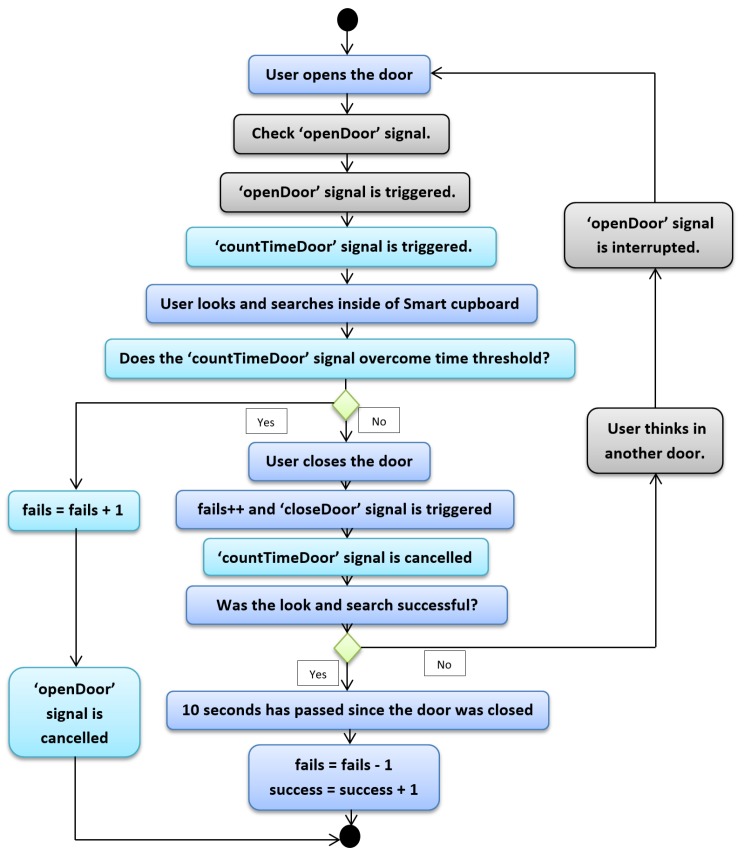
Python script—Third case.

Figure 6 depicts the third case. The beginning is the same as aforementioned cases, only with the difference that another signal is triggered when the door is opened by the user; this signal is called “countTimeDoor” (we have not mentioned the signal before in order to avoid over-long explanation, but it was also present in the previous cases). The signal’s aim is to start to count the time that an SC door is held open. This period of time matches with period of time the user is searching an object inside of the SC. The assigned threshold for this signal was 10 s, that is, the user has 10 s to find the item, and if this time is surpassed the system increases the fail counter. We determined the threshold of the “countTimeDoor” signal in the same way as we determined the 10 s in order to know whether user was successful (i.e., through experiments made by ourselves and other users), until we were able to determine a proper threshold. We assume that the user overcame the signal threshold, so the fail counter increases. Furthermore, the “openDoor” signal is canceled, since if it were not so, when the user closed the door again, our system would increase the fail counter once again. Finally, it is worth mentioning that if the user closes the door before 10 s, the “countTimeDoor” signal is canceled in order to avoid increasing the fail counter twice.

**Figure 7:**
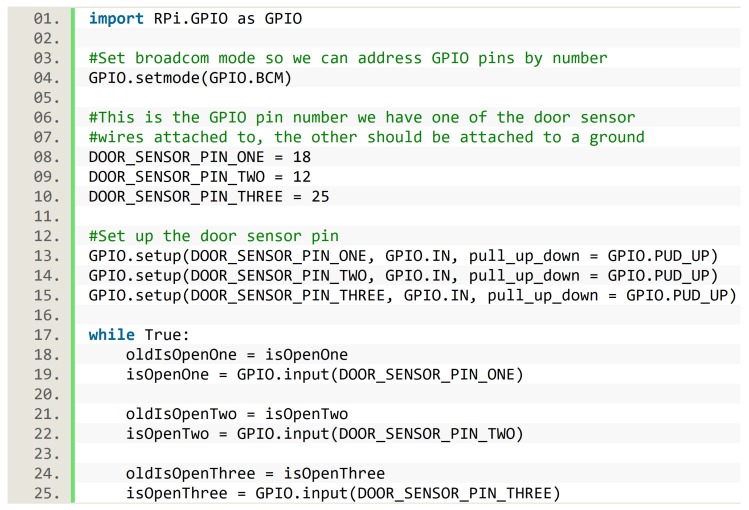
Implementation details about pins in the Python script of the smart cupboard.

The algorithm was written in Python, and for the sake of reproducibility, we describe how the implementation of this algorithm handled the pins. In order to manage pins inside the script, we used the RPi.GPIO library. There were two options to enumerate the pins. The first was GPIO mode, in which each pin had the same number as their physical position, hence pins were enumerated from 1 to 40. The second option was BCM (Broadcom mode), in which pins were numerated in order to match with Broadcom chip, which was the CPU (central processing unit) of the Raspberry. We used BCM for the implementation of the presented algorithm. The algorithm implementation also needed to set up some pins as input pins, and this was achieved with the predefined function GPIO.setup. Finally, the GPIO.input function provided the current state of each door sensor, true when the two pieces of a door sensor were separated (i.e., the door was open), and false otherwise. Figure 7 presents an excerpt of the Python implementation used in the SC, showing the aforementioned implementation details about pins. Door sensor states were used as previously described when presenting the algorithm.

## 4. Experimentation

### 4.1. Participants

We recruited 23 people for participation in this user study. Participants were 36.17 years old on average (SD = 12.80) in a range from 18 to 60 years old, and had studied for 14.86 years on average (SD = 2.88). Among the participants, only 8.69% were studying or work in computer science. Males comprised 39.13% of participants. Participation in this experiment was voluntary and unpaid.

### 4.2. Procedure

In order to evaluate whether the SC is able to measure memory, several tests were conducted. In the first test, a user was asked to observe the inside of the SC. The user had to memorize certain items inside it in the acquisition phase. Then, the user was asked to find certain items in the retrieval phase. A test of face–name pairs [17] was used as a control method, since this kind of test has been proven to measure memory. The test consists of showing a list of face–name pairs, so the user memorized them in the acquisition phase, for later selection of the name associated with each face in the retrieval phase. The main goal was to determine whether both results were correlated, besides performing other analyses concerning the relation of the results with the participant features.

**Figure 8:**
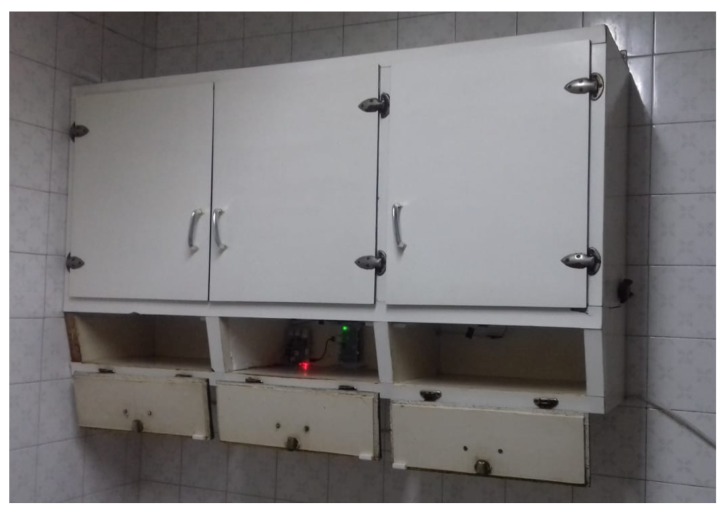
Smart cupboard.

The test was conducted in a real kitchen so that participants were familiar with the scenario. The Raspberry was set up inside a kitchen cupboard as shown in Figure 8. The size of this cupboard was 130 cm width × 71 cm height × 29.5 cm depth, and it was 150 cm above the ground. In spite of having five compartments, we only used the bottom three in order to avoid having compartments of different sizes.

To assess the memory of participants with the SC, we selected 30 different items. All these items were typical objects commonly found inside cupboards, and these objects were: a cup, a sweet corn can, a chili can, an egg, a box of matches, an evaporated milk carton, a soda, a bag of breadcrumbs, a beer can, a jar of chili peppers, a potato, a jar of lentils, a can of olives, a jar of mayonnaise, a carton of chocolate milkshake, a can of grapes, a jar of soup cubes, a can of peaches in syrup, a can of condensed milk, salt, a box of baking powder, a can of green peas, a milk bread, a jar of jam, a teaspoon, a jar of sausages, honey, a can of tuna, a bag of tea, and a jar of oregano. In this experiment, each participant followed the same process. The experimenter introduced the steps briefly to each user. In the acquisition phase, the experimenter asked each user to memorize all the items of each compartment, and they had 30 s per compartment—1 min and 30 s in total.

In the retrieval phase, the experimenter told the participant that he would ask them to find objects selected in a random order from the ones inside the cupboard and previously memorized by them. Thus, the participant had to open the compartment where they thought each required object was. Furthermore, the experimenter indicated that only one door could be open at a time (i.e., before the participant opened another door, they had to close the current door). Once the participant had found the required object, grabbed it from the SC, and closed the door, the experimenter asked questions about the item. The questions were varied and related to cooking or eating. For instance, what recipes or dishes would you cook with this object? Or, what time of day do you usually eat this product? Or, do you think this product is healthy and why or why not? And so on. The reasons for these questions were to delay the retrieval phase and to increase the difficulty of the test. When users opened the door in order to find an object, they could re-memorize where each object was (reinforcing the initial learning), since it was unavoidable to let them see the contents again. Thus, the goals of these distracting questions were to compensate for this aspect and to be more similar to realistic daily conditions.

This process was repeated with ten different objects for each user. Due to the size of SC and the amount of items, two rounds were required. In each round, each participant had an acquisition phase in order to memorize the content of compartments concerning 15 objects, and had a retrieval phase to sequentially find 10 objects. When the first round was finished, the experimenter asked the user to leave the kitchen. While the participant was outside the kitchen, the experimenter set up the second round, and then invited the participant to come into the kitchen again. Hence, users performed 20 memory retrievals about 20 different objects. In this way, this memory test had an accuracy error margin of 5%, which is considered as appropriate in memory tests [18]. We decided that all participants had the same conditions; hence, before starting this procedure, a list of items was selected for use by all participants.

The list had 15 items for the first round and 15 items for the second one. The order of objects was selected randomly, because we wanted to avoid the case where objects were organized by size or semantic categories, in order to avoid bias in the memory measurement due to different memorization techniques. Table 2 indicates the order of objects in the SC, distributed in compartments per round. Furthermore, another requirement of the experimentation phase was that objects could not be behind others (i.e., all objects had to be visible from the participant’s position). The experimenter was always with the participants, except when they were outside the kitchen. The experimenter made sure that the participants properly followed the experimentation protocol. For instance, the experimenter was advised to take note if any participant closed the door twice because it had not been closed with enough strength the first time. In this case, the system would register an additional fail, so then we could revise the logs to know what had happened.

**Figure 9:**
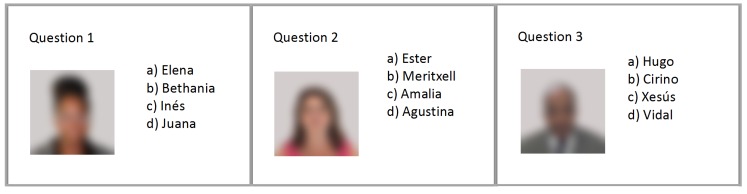
Test of face–name pairs.

The next step for participants was to take a control test based on face–name pairs in order to statistically compare these results with the SC results and to determine whether our SC is able to measure memory. This test was similar to the common memory tests about face–name pairs in the literature [17,19], and we used a short-time version that did not require several days for acquisition and retrieval phases. In this way, each participant could do all the experimentation in the same day, facilitating the task of recruiting unpaid volunteer participants. In this experiment, the test of face–name pairs consisted of showing a series of 30 face–name pairs to the participant such that they memorized these associations in the acquisition phase. Each face–name pair was presented to the user for 6 s, and consequently the whole acquisition phase took 3 min. In the retrieval phase, each participant had to respond to 30 questions. Each question was composed of a face image and four name options, and the participant had to fill a form provided by the experimenter with the answers to these questions. Figure 9 shows an example of three questions. The face images have been blurred in this article to protect the privacy of the models. The retrieval phase had no time limit, but the experimenter instructed the participants to reply to the questions as accurately and quickly as possible, and the reaction time was measured.

Furthermore, the participants engaged in a self-reported memory test. We performed this test in order to compare the SC results with other memory-related variables. Since the self-assessment of memory has proved to be relevant for evaluating memory despite other influencing factors such as personality [20], we included this brief self-reported memory test as another control test. We selected a short self-reported test available from the Psychology Today website (http://psychologytoday.tests.psychtests.com/bin/transfer?req=MTF8MzM2MHw2NzI5MDI0fDB8MQ==) for its brevity and its simplicity. In this test, participants replied to seven questions with a five-point Likert scale. The questions of this test were: (1) Do you have difficulty in remembering people’s names or phone numbers? (2) How often do you find yourself trying to remember the location of everyday items (e.g., your keys, wallet, glasses, etc.)? (3) How often do you have to replace passwords (numerical or verbal) because you’ve forgotten the original one? (4) How often do you find yourself asking questions like, “What was I about to do next?” (5) How often do you end up arranging overlapped plans because you forgot you had made previous plans with someone else? (6) How often do you have to ask someone to repeat instructions or a story because you can’t remember what was said the first time? (7) How often do you have difficulty in remembering where you parked your car? The last question was only replied by participants who had a driving license.

The experimenter also asked participants to reply a brief demographic test to extract the information presented in Section 4.1 when introducing the sample of participants. Once we finished all the experimentation with all the participants, we analyzed the obtained data as described in the next section.

## 5. Results

We performed several analyses considering the memory measurements in the different methods, the reaction time, and the age of participants. Firstly, we compared the memory measurement results between the SC test and the face–name test, reporting the accuracies of participants in the retrieval phase.

In order to double-check that the sensors system of the SC was working properly, the experimenter took notes about the fails and successes of participants during the experimentation phase, and then the notes were contrasted against the system log. The notes and SC logs and results matched perfectly. The accuracy percentage of each participant was calculated as shown in Equation (1) as a measurement of their memory:

(1) $$a=\frac{s}{s+f}\cdot 100,$$

where *a* is the accuracy percentage, *s* is the number of successes, and *f* is the number of fails.

The accuracy of each participant in the retrieval of face–name pairs was calculated in a similar way. The experimenter checked the final test results, and the percentage was calculated as shown in Equation (2):

(2) $$a=\frac{s}{n}\cdot 100,$$

where *a* is the accuracy percentage, *s* is the number of successes, and *n* is the total number of questions.

**Figure 10:**
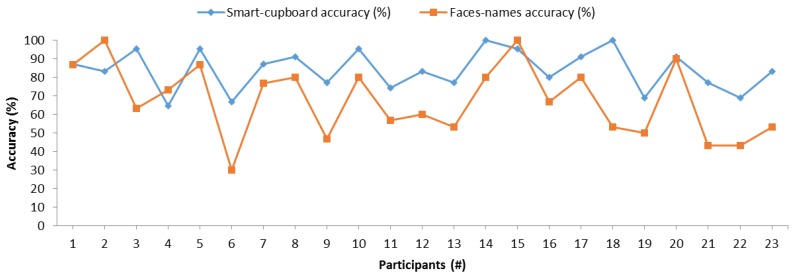
Comparison of memory measurements between the smart cupboard (SC) and the face–name pairs test.

Figure 10 compares the memory accuracies of the SC and face–name pairs, and one can observe that both measurements methods followed similar trends and shapes. Thus, there may be a correlation between these measurement methods. In order to statistically and reliably corroborate this correlation, we conducted a Pearson’s correlation test between the results of the two memory measurement methods. Table 3 shows the results of this correlation test. According to the Pearson correlation coefficient [21], both memory measurement methods had a significant positive correlation. This positive correlation was confirmed with the Kendall’s tau coefficient of 0.470 with a *p*-value of 0.003 and Spearman’s rho coefficient of 0.620 with a *p*-value of 0.002. The correlation between the SC test and the test of face–name pairs proves that our SC sensors system is able to measure memory, since the control memory measurement method has already been scientifically validated.

**Figure 11:**
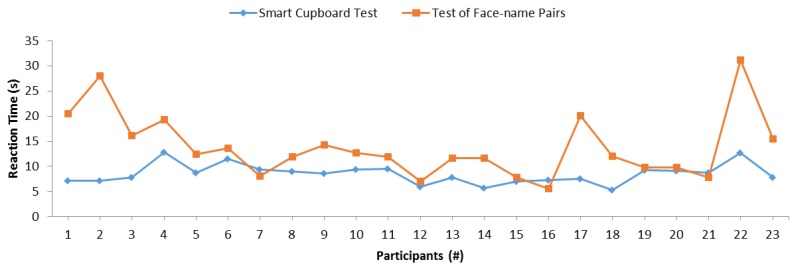
Comparison between the SC reaction time and the reaction time of the face–name test.

Moreover, we analyzed the reaction time of participants in the SC and face–name pairs tests. Figure 11 depicts the reaction time of each participant in both tests. The blue line represents the SC test and the orange line represents the test of face–name pairs. In order to calculate the reaction time of the SC test, the experimenter took note of the time spent by a participant to remember each item inside the SC. Then, this was compared with the time in the system log in order to check that all times were correct. Thus, 20 reaction time results were obtained for each participant. Finally, the reaction time of each participant was calculated as the mean of all obtained times. In the face–name test, the experimenter also measured the time spent by a participant in order to perform the test. The reaction time was obtained from the division of the total time by the total number of questions by each participant. According to the trend and direction of both lines in the graph, it is not easy to appreciate the similarity in general. Nonetheless, a certain similar behavior is appreciated between participant 7 and participant 15. A similar correlation can be appreciated between these two tests, since both lines are almost parallel. Because this observational analysis is not sufficient to determine whether there was any significant relation between tests in reaction times, we performed another Pearson’s correlation test to statistically determine whether there was a statistically significant correlation. Table 4 depicts the result of the correlation test. It shows that both variables were not significantly correlated. Neither Kendall’s tau coefficient nor Spearman’s rho coefficient detected any significant correlation with respective *p*-values of 0.597 and 0.428. Thus, in these experiments, the reaction time of our SC test did not correlate with the reaction time of the control test of face–name pairs.

**Figure 12:**
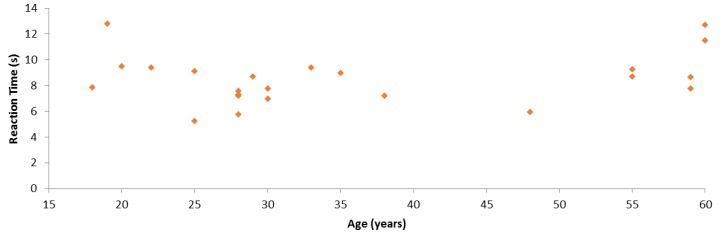
Comparison between the reaction time in the smart cupboard test and participant age.

We also conducted an analysis of the relation between participant age and SC reaction times. Figure 12 represents each participant considering these two aspects. It is worth highlighting that we asked people to participate in these experiments, considering their age, to have both young and aged people. In particular, there were six young participants in the 18–25 years old range and six aged participants in the 55–60 years old range. In order to determine whether there was a statistically significant correlation, we performed three correlation tests between SC results and age. Table 5 shows the results of the Pearson’s correlation test. One can observe that these variables were not correlated according to this test. In addition, neither Kendall’s tau coefficient nor Spearman’s rho coefficient detected any significant correlation, with respective *p*-values of 0.265 and 0.300. Thus, SC results and age were not statistically significantly correlated in these experiments.

We also performed an analysis comparing the accuracy of participants in the SC test and the results of the self-reported memory test. Each possible answer of the self-reported memory test among the options “almost always”, “often”, “sometimes”, “rarely”, and “almost never” were respectively assigned the values 0, 1, 2, 3, and 4. The results of this test were standardized as a percentage calculated with Equation (3):

(3) $$r=\frac{\sum_{i=1}^{n}x_{i}}{n}\cdot \frac{100}{V_{max}},$$

where *r* is the result of the self-reported test, $x_{i}$ is the value of each answered question, *n* is the total number of questions, and $V_{max}$ is the maximum value that a response can have (i.e., 4 in this case).

**Figure 13:**
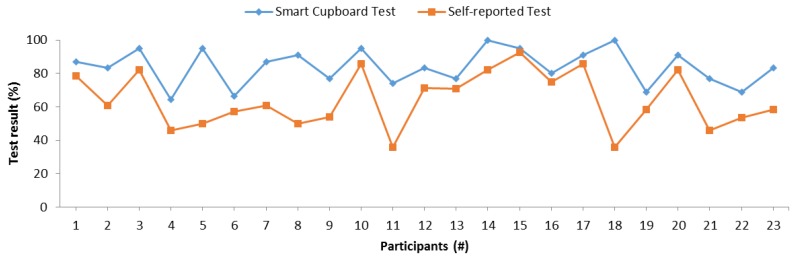
Comparison between the accuracy of SC and that of self-reported tests.

Figure 13 shows the accuracy of the SC test and this self-reported test. There were similarities between both tests in some cases, as one can observe in the intervals between participants 1 to 4, participants 9 to 11, and participants 15 to 17. To determine the statistical significance of this relation, we conducted a Pearson’s correlation test, and Table 6 presents the results. The test indicated that the memory results between the SC test and the self-reported one were significantly correlated. This correlation was confirmed with the Kendall’s tau coefficient of 0.383 with a *p*-value of 0.014 and Spearman’s rho coefficient of 0.451 with a *p*-value of 0.031. The self-reported test is a subjective test, and personality may cause bias in the results, since this test actually measures self-perceived memory rather than actual memory (calculated as the accuracy retrieving information previously acquired). According to these experiments, the SC results were correlated with self-perceived memory, despite the possible bias because of personality.

## 6. Discussion and Conclusions

The article proposed a new mechanism of measuring memory with the SC as a novel IoT sensors system. We presented the design of the SC as a cupboard with three sensorized doors with magnetic door sensors connected to Internet via a Raspberry Pi 3B board with the corresponding software for evaluating user memory.

The main goal was to have a device able to assess the memory in a familiar environment without requiring additional effort from the user. Thus, we presented a solution in which the memory measurement can be continuous and based on normal routine. The results based on 23 participants in a wide age range (18 to 60 years old) showed that the accuracy of participants in finding objects in the SC in a controlled environment was statistically significantly correlated with the accuracy of participants in retrieving face–name associations in a validated type of memory test. The accuracy of the SC test was also statistically significantly correlated with a self-reported memory test.

The current work attempted to find a solution that was low-cost as possible, in order to propose a step towards a solution that can get to the market with a profitable margin, so that enterprises may be interested and our solution can make a real impact on society. Note that the most fundamental components of this solution were the magnetic door sensors, and in particular we used a door sensor model that only cost $2.46. The Raspberry Pi 3B board could be easily replaced by any other low-cost/green processing board in the market by using the same software and adapting the input pins. Thus, a very cheap solution could potentially be developed for converting a cupboard into a SC, so the user could install the sensor systems of their cupboard without needing to replace their original cupboard.

In the early detection of diseases, it can be difficult to sell products to healthy people, even if the product is cheap. We argue that as in the case of many other successful smart devices in the market (e.g., smartphones, smartbands, and smart TVs), SCs could have multi-purpose functionalities for successfully getting to the market. In this line, the SC could also be useful for detecting eating patterns by classifying different kinds of food in different compartments. Eating patterns can be useful for controlling dietary habits to reduce obesity, which is an issue for many people in countries like the US [22]. Eating patterns could also be useful for tracking emotions by considering their known relationship [23].

The experiments showed that the reaction time measured by the SC test did not correlate with the reaction of the control test about face–name pairs. However, these results are not conclusive, since the number of participants (*n* = 23) was not sufficient to detect medium effect sizes according to the analysis based on statistical power performed by the G*Power 3 tool [24]. In addition, not all memory tests need to have a correlation between reaction time and memory. In fact, strictly a memory test needs to provide some measure that correlates with memory, which could be either accuracy or reaction time, but not necessarily both. Thus, the proposed SC test is a reliable memory test according to the common standards of memory tests [25].

In addition, the accuracy of the SC memory test correlated with self-perceived memory, even though the literature supports that self-perceived memory is influenced by factors such as personality [20], which could lead to differences between self-perceived memory and actual memory.

One limitation of the current version of the SC is that it is based on the assumption that there is only one person using the SC to reliably measure their memory. We plan to overcome this limitation by including an identification mechanism, which could either be (1) facial identification with a low-cost camera following our previous work in facial authentication [26] or (2) radio-frequency identification, which would require the user to carry a card for the identification. We will select one of these options considering economic aspects, technological reliability, and user experience. In this manner, the SC will determine who is using it and perform different measurements for the different family members. In general, the inclusion of identification will allow engineers to develop SC applications for providing customized services to the user.

In the future, we plan to conduct a study with Alzheimer’s patients over a 10-month period to detect memory losses during the evolution of this disease, by measuring the memory of the same persons through the study with the proposed SC-based approach. If possible, we will also enroll people considered to probably start having Alzheimer’s disease soon, known by the analysis of genetic information in descendants of people with Alzheimer’s. As a control group, we also plan to track the memory evolution of a group of healthy people, which may include some of the participants of the presented study. In this way, we plan to detect improvement opportunities and further assess whether it is possible to track memory losses and to detect Alzheimer’s at an early stage.

Another future work is the development of an app whose main aim will be to explain to users how to turn a normal cupboard into an SC using gamification to overcome the barrier of a possible difficult installation. Finally, our efforts will focus on improvement of energy efficiency, since cupboards are not used very frequently. This could be achieved by lowering the checking frequency of sensors when users do not usually use them, based on an initial training phase, following a similar approach to our previous one in green communications with smartbands [27]. Another option that involves the power system is to use some energy-harvesting techniques to overcome the limitation of the long-term use of the SC powered by batteries. The aim of harvesting techniques is to accumulate energy from several sources that capture energy from the environment. Once the energy is accumulated, it can be used in the SC to track user memory. Several examples of energy harvesting can be found in the literature [28], but techniques that involve components such as micro-photovoltaic cells, micro-thermoelectric generators [29], or indoor ambient light [30] may be the most suitable for SCs. Furthermore, we also plan to develop an app for remotely consulting memory measurement results from any mobile device, and to provide notifications if a family member is starting to have significant memory losses.

## Figures and Tables

**Table 1:** Comparison between the current work and the most closely related ones.

Question	Current Approach	Nonavinakere et al. [8]	Crema et al. [9]	Narendiran et al. [10]	Ishii et al. [11]	Paul et al. [14]
Does this work use IoT?	✓	-	-	-	✓	
Does this work use Raspberry Pi?	✓	✓	✓	✓	✓	✓
Does this work present a low-cost solution for health monitoring?	✓	✓	-	-	-	✓
Does this system use wearable devices?	-	✓	✓	-	-	✓
Can this solution be applied without qualified staff?	✓	-	-	-	✓	
Does this solution measure memory?	✓	-	-	-	✓	
Does this solution measure cardiac measures? (heart rate, heart rate variability)	-	-	-	-	-	✓
Does this solution measure temperature?	-	-	-	-	-	✓
Does this solution have the potential to measure any health indicator by just analyzing the daily activities of users?	✓	-	-	✓	✓	✓
Does this solution have the potential to measure memory by just analyzing the daily activities of users?	✓	-	-	✓	✓	
Could this solution help to detect memory-impairment diseases at an early stage?	✓	-	✓	✓	✓	

**Table 2:** Order of objects in the experimentation with the smart cupboard.

Object	Compartment	Round	Object	Compartment	Round
Cup	First	First	Grapes	First	Second
Sweet Corn			Soup cubes		
Chili			Peaches in syrup		
Egg			Condensed milk		
Box of Matches			Salt		
Evaporated milk	Second		Baking powder	Second	
Soda			Green peas		
Breadcrumb			Milk bread		
Beer			Jam		
Chili peppers			Teaspoon		
Potato	Third		Sausages	Third	
Lentils			Honey		
Olives			Tuna		
Mayonnaise			Tea		
Chocolate milkshake			Oregano		

**Table 3:** Correlation between the accuracy of the SC and the accuracy of the face–name test.

		Accuracy Smart Cupboard	Faces-Name Test
	Pearson Correlation	1	0.597 **
Accuracy Smart Cupboard	Sig. (2-tailed)		0.003
	*N*	23	23
	Pearson Correlation	0.597 **	1
Faces-Name Test	Sig. (2-tailed)	0.003	
	*N*	23	23

**. Correlation is significant at the 0.01 level (2-tailed).

**Table 4:** Correlation between the SC reaction time and the reaction time of the face–name test.

		Reaction Time Smart Cupboard	Reaction Time Face-Name Test
	Pearson Correlation	1	0.341
Reaction Time Smart Cupboard	Sig. (2-tailed)		0.111
	*N*	23	23
	Pearson Correlation	0.341	1
Reaction Time Face-Name Test	Sig. (2-tailed)	0.111	
	*N*	23	23

**Table 5:** Correlation between the reaction time and participant age in the smart cupboard test.

		Age	Reaction Time Smart Cupboard
	Pearson Correlation	1	0.306
Age	Sig. (2-tailed)		0.156
	*N*	23	23
	Pearson Correlation	0.306	1
Reaction Time Smart Cupboard	Sig. (2-tailed)	0.156	
	*N*	23	23

**Table 6:** Correlation between the accuracy of SC and that of self-reported tests.

		Smart Cupboard	Accuracy Self- Reported Test
	Pearson Correlation	1	0.443 *
Smart Cupboard	Sig. (2-tailed)		0.034
	*N*	23	23
	Pearson Correlation	0.443 *	1
Accuracy Self-Reported Test	Sig. (2-tailed)	0.034	
	*N*	23	23

*. Correlation is significant at the 0.05 level (2-tailed).

## Abbreviations

The following abbreviations are used in this manuscript:

GPIO	General-Purposes Input/Output
I2C	Inter-Integrated Circuit
ID	Identifier
IoT	Internet of Things
M2M	Machine-to-Machine
SD	Standard Deviation
SPI	Serial Peripheral Interface
SC	Smart Cupboard
TV	Television
UART	Universal Asynchronous Receiver-Transmitter
US	United States
